# Physiotherapy-led, community-based airway clearance services for people with chronic lung conditions: a retrospective descriptive evaluation of an existing model of care

**DOI:** 10.1186/s12913-024-10550-x

**Published:** 2024-01-18

**Authors:** Laura Cooper, Kylie Johnston, Marie Williams

**Affiliations:** 1 Innovation, IMPlementation And Clinical Translation in Health (IIMPACT), University of South Australia, Allied Health and Human Performance, North Terrace, Adelaide, 5000 Australia; 2 Southern Adelaide Local Health Network, Respiratory GP Plus Out of Hospital Services, Noarlunga GP Plus Super Clinic, Alexander Kelly Drive, Adelaide, South Australia 5168 Australia

**Keywords:** Airway clearance, Respiratory, Physiotherapy, Chronic lung conditions, Service provision

## Abstract

**Objectives:**

Airway clearance interventions are recommended for people with chronic lung conditions and mucus hypersecretion, but there are few published models of care or descriptions of airway clearance service provision. This evaluation describes a dedicated, physiotherapy-led, community-based airway clearance service in a metropolitan local health network.

**Design:**

Retrospective evaluation using existing airway clearance service administrative database.

**Participants:**

All first referrals to the airway clearance service in a 5-year period (1/1/2017 to 31/12/2021).

**Main outcome measures:**

Available service data grouped into four domains: participant demographics, referral demographics, service provision and outcomes.

**Results:**

Of the 1335 first referrals eligible for inclusion, 1157 (87%) people attended. Bronchiectasis was the commonest condition (*n* = 649/1135, 49%). A total of 2996 occasions of service (face to face clinic *n* = 2108, 70%, phone *n* = 736, 25%, telehealth *n* = 99, 3%, home visit *n* = 53, 2%) were delivered. Airway clearance devices frequently prescribed were the Aerobika (525/1157, 45%), bubble-positive expiratory pressure (263/1157, 23%) and the Acapella (127/1157, 11%). On average, initial appointment with the airway clearance service occurred within 36 days of referral and people attended the service three times. Individuals voluntarily completed both pre/post service questionnaires around a third of the time. At least half of responders reported an improvement in respiratory symptom outcome measures consistent with the minimum clinically important difference.

**Conclusions:**

This evaluation describes an airway clearance service as it exists, providing an example from which airway clearance services can be planned, implemented and improved.

**Supplementary Information:**

The online version contains supplementary material available at 10.1186/s12913-024-10550-x.

## Introduction

Chronic lung conditions are estimated to affect 545 million people worldwide and have a significant impact on the health care system, economic expenditure, and people’s lives [1]. Three of the most prevalent and burdensome lung conditions, asthma, chronic obstructive pulmonary disease (COPD) and bronchiectasis, have common clinical symptoms of mucus hypersecretion, coughing and breathlessness [2–4]. More recently, bronchiectasis and COPD are included in the group characterised as muco-obstructive lung disease [5].

Clinical guidelines for these chronic lung conditions recognise the role of physiotherapy in the management of mucus hypersecretion [6–11]. For example, European Respiratory Society guidelines for management of adults with bronchiectasis suggest people with difficulty clearing sputum should be taught an airway clearance technique by a trained respiratory physiotherapist (weak recommendation, low quality evidence) [10]. In the Thoracic Society of Australia and New Zealand 2023 position statement, “ACTs are recommended. These should be individualised, and their method and frequency reviewed, preferably biannually by a respiratory physiotherapist.” (6 p345). Physiotherapy-led airway clearance assessment and interventions typically include education, personalised regimens of breathing exercises and clearance strategies to facilitate effective expectoration of excess lung secretions [12]. This therapy may be applied as an intervention for individuals [13] or as a desirable component of pulmonary rehabilitation (PR) programs [14]; in hospital inpatient settings [15, 16], or in outpatient clinics [17]. A survey of 91 Australian providers reported that airway clearance services (ACS) in non-acute settings lacked dedicated funding, resources, and staff, and in non-metropolitan areas, had fewer experienced clinicians, inadequate administrative support, and lower rates of device prescription at higher costs to clients [18]. Published accounts of ACS models of care in non-acute settings are rare, in contrast to the breadth and depth of information underpinning service provision and evaluation in PR [19] and specialised cystic fibrosis (CF) care [20, 21].

This retrospective audit aimed to describe and evaluate the first five years of a dedicated, physiotherapy-led, community-based ACS in a metropolitan local health network, in South Australia. The objectives of this study were to describe service users; evaluate implementation of the planned service model (process evaluation) and report on service effects (impact evaluation) according to the theoretical framework, the PRECEDE PROCEED Model [22]. Conducting and reporting this audit may support future planning of equitable access for participants, inform staffing decisions, and choices of assessment tools and clinical intervention strategies for this service and potentially other airway clearance services.

## Methods

### Evaluation context and design

In 2016 a metropolitan local health network, the Southern Adelaide Local Health Network (SALHN) identified a need to reduce the number of hospital admissions associated with exacerbations of chronic lung disease and to transition appropriate hospital-based outpatient services to community settings. One of the key objectives to meet this need was to develop a dedicated, physiotherapy-led, ACS in the community to assist people with chronic lung conditions to self-manage their cough and mucus hypersecretion and improve their health-related quality of life. The service plan was to provide most occasions of service on-site in a face-to-face community clinic. The capability to provide the service in a client’s home or via phone/telehealth platforms was available and encouraged when client frailty prevented clinic-based care.

Ethical approval for this retrospective evaluation was sought and approved by the Southern Adelaide Clinical Human Research Ethics Committee (HREC) (27/9/2022, ID:175.22) and the University of South Australia HREC (12/10/2022, ID:205,044).

### Health service context

SALHN provides care for more than 378,000 people [23]. One major tertiary institution provides hospital-based care for this region, around which rehabilitation and community service hubs are based. Prospectively, workforce planning for this ACS designated one full time equivalent (FTE) respiratory physiotherapist to operate a dedicated weekday clinic (at set days/times) from within these community hubs. The service aimed to provide education on the role of airway clearance in self-management, initiate airway clearance strategies/prescribe devices for suitable participants and address any associated respiratory symptoms through referral to relevant allied health disciplines and respiratory nursing services that were co-located on site. The ACS was publicly funded with no costs for service or devices to the consumer.

**Fig. 1:**
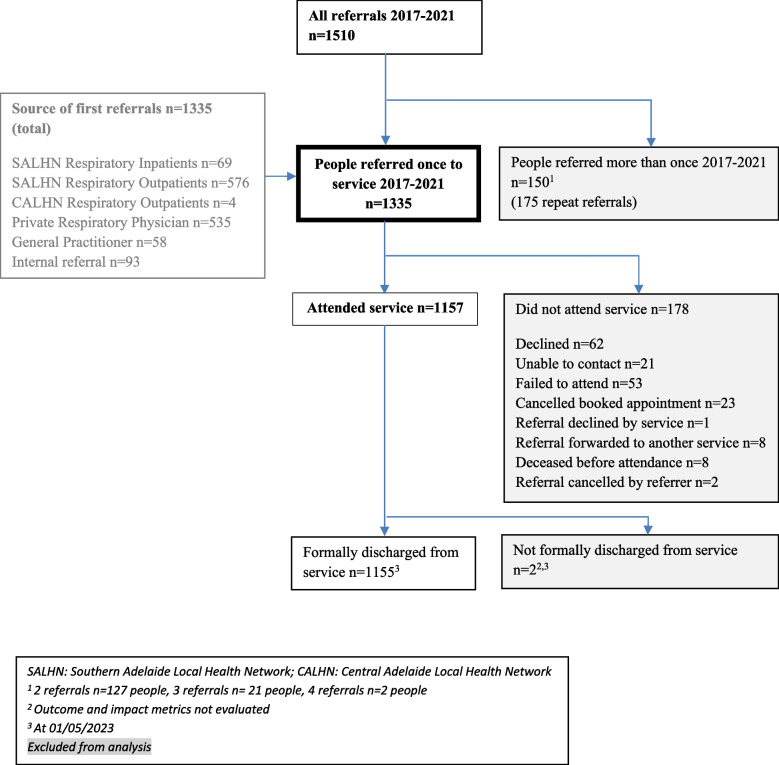
Referral sampling frame

While the service and routine service data collection officially started on 26/6/16, for this retrospective audit, data was included from clients who attended for the first time during the first five complete calendar years of the service. All adults with a chronic lung condition referred to the ACS for the first time between 1/1/2017 and 31/12/2021 were eligible for inclusion within this evaluation. Repeat referrals for these included individuals and any associated data were excluded from analysis. Referrals were accepted from public and private physicians, nurses and allied health professionals and were grouped into referral source categories (Fig. 1). Triage categories of referral urgency were pre-determined (1 = 0–30 days, 2 = 30–60 days, 3 = 60 + days) and were based on the information provided on the referral and/or the electronic medical record. Decisions about which data would be collected and how were made as part of the planning phase of the service. Patient-reported outcome measures (PROMs) were prospectively chosen by the service key stakeholder group (including health professionals from physiotherapy, nursing, medical and general practice disciplines) and in accordance with other local integrated services (PR, GPs) to reduce participant burden and duplication. The choice of PROMs was based on suitability and validity in older people with chronic lung conditions anticipated to be seen by the service [24–27], and the availability of the PROMs free of charge. Service-specific forms (registration and satisfaction surveys) were prepared before the service was implemented. While this ACS was open to referrals for adults with any chronic lung condition, people with CF and motor-neuron disease were not referred as they were managed by specialised state-wide disease specific services.

### Data source

Data collected for each person eligible for inclusion in this audit was entered on to a spreadsheet by a single administrator (Table 1). Referral and participant demographic data were recorded from the paper referral and the electronic medical record. The individual’s diagnosis or condition and reasons for referral were transcribed verbatim from the referral. Individuals were posted registration forms and PROMs appropriate to their specific health condition. All referred individuals received a Leicester Cough Questionnaire (LCQ), a Depression, Stress and Anxiety Scale-21 (DASS21) and an ACS registration form. The ACS registration form was purpose designed for participants to self-report symptoms (sputum quantity, colour, viscosity) and associated co-morbidities (reflux, sinusitis) and bring to their first appointment. Those with COPD and/or bronchiectasis received the COPD Assessment Tool (CAT) and/or the Quality of Life – Bronchiectasis (QOL-B) questionnaire respectively.

**Table 1:** Airway clearance service data capture

Service Metrics	Point of service delivery when data is collected				Target participants			Data source			Data collection method	
	Referral	Booking	Initial	Discharge	All	COPD	BE	Referral	EMR	Post	Routine	Voluntary
Number of referrals	✓				✓			✓			✓	
Date of referral	✓				✓			✓			✓	
Referral source	✓				✓			✓			✓	
Date of birth (age)	✓				✓			✓			✓	
Gender	✓				✓			✓	✓		✓	
Diagnosis	✓				✓			✓			✓	
Postcode	✓				✓			✓	✓		✓	
Initial appointment date		✓	✓		✓				✓		✓	
Time to first appointment			✓		✓				✓		✓	
On-referrals made^b^				✓	✓				✓		✓	
Occasions of service				✓	✓				✓		✓	
Mode of delivery				✓	✓				✓		✓	
Length of time in service				✓	✓				✓		✓	
Discharge letter sent				✓	✓				✓		✓	
Self-reported symptoms			✓		✓					✓		✓
LCQ [23]			✓	✓^a^	✓					✓		✓
DASS 21 [22]			✓	✓^a^	✓					✓		✓
CAT [21]			✓	✓^a^		✓				✓		✓
QOL-B [24]			✓	✓^a^			✓			✓		✓
Satisfaction survey				✓	✓					✓		✓

*EMR* Electronic Medical Record, *LCQ* Leicester Cough Questionnaire, *CAT* COPD Assessment Tool, *DASS 21* Depression Anxiety and Stress Scale, *QOL-B* Quality of Life – Bronchiectasis, *COPD* Chronic Obstructive Pulmonary Disease, *BE* Bronchiectasis

^a^Post questionnaires sent if pre questionnaires completed

^b^Recorded any referrals made to other health professionals during the client’s engagement with the ACS

**Fig. 2:**
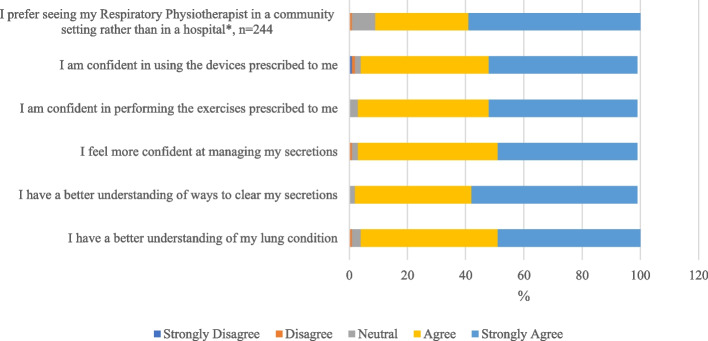
Airway clearance service participant satisfaction survey, *n *= 289 (*statement added in 2018)

Care was individualised and there was no predetermined limit to the number of sessions. Where possible, goals of care were agreed between the treating physiotherapist and the participant at the first appointment. The choice of airway clearance technique, device prescription and/or lifestyle modification strategies employed were based on clinical need/effectiveness, adherence to the therapy, and client preference. Symptoms such as incontinence, musculoskeletal pain and reflux were addressed on an individual basis with on-referral to other health professionals as required. Participants attended the program until the agreed goals of care were achieved. Upon formal discharge from the service, follow up PROMs (for participants completing them prior to the service) and a satisfaction survey were sent by post with a reply-paid envelope for return (Supplementary Data, S1). The satisfaction survey included six statements (using a 5-point Likert scale) and free text feedback options inviting people to report on the service (Fig. 2). Participants voluntarily completed PROMs at home and returned them to the site (at initial face-to-face appointment) or via post (after discharge). The treating clinician scored the PROMs and included the results in the medical documentation as appropriate. The treating physiotherapist produced a discharge summary for the participant’s general practitioner (GP) and referrer.

### Data management and quality control

The variables of interest in this descriptive analysis were grouped into domains: referrals (source, date); participants (demographic characteristics); service provision (wait times, occasions of service, modes of delivery); outcomes (PROMS, satisfaction) (Table 1). Measures of socioeconomic status (Index of Relative Socio-Economic Advantage and Disadvantage (IRSAD) [28] and accessibility/remoteness of participants (Accessibility/Remoteness Index of Australia (ARIA) [29] were derived from participants’ residential postcodes. The frequency and nature of missing data was reviewed: where possible missing hospital system data were retrieved from alternate platforms. PROMs and satisfaction surveys relied on voluntary participant completion and were not routinely checked for missing items or discussed with the participants (Table 1). PROM scores were calculated as recommended by the instrument developers [24–27]. No imputation was used for missing data.

### Data analysis

The sampling frame considered all persons referred to the ACS and was subdivided into first-time referrals (included in evaluation analysis) and repeat referrals (excluded from all subsequent analysis). Differences (in demographics, referral source and referral-stated condition) between those referred who did and did not participate in the service were explored using chi squared tests (α = 0.05). For people who participated in the ACS (i.e., attended at least one appointment) descriptive statistics (frequencies, percentages, central tendency and spread) were used to report all categorical and continuous data on demographics, ACS service provision and self-reported symptom information.

The response rate of completed pre- and post- service PROMs was reported as a percentage of the total number of participants sent them by post. Where available (i.e., questionnaires voluntarily returned via reply-paid envelopes), paired (pre-post) PROM data for individuals was explored for clinical significance according to the minimal clinically important difference (MCID) reported for each instrument [24–27]. Pre-post changes for individuals reaching the MCID for the LCQ (+ 1.3 point change [26]), DASS (-5 point change [25]), CAT (-2 point change [24]) and QOL-B (respiratory domain ≥ 8.0 points [27]) were interpreted to indicate an improvement in clinical condition. The number of individuals reaching the MCID for each PROM was reported and expressed as a proportion of the number of the paired PROM questionnaires returned. The satisfaction survey response rate was reported as a percentage of the total number of participants who were discharged from the service and survey responses were reported descriptively.

## Results

A total of 1510 referrals were received between 2017 and 2021. First-time referrals for individuals (*n* = 1335/1510) were included in the evaluation and repeat referrals (*n* = 175/1510) for these individuals between 2017 and 2021 were excluded. Eighty-seven percent of first-time referred individuals (1157/1335) attended the ACS (Fig. 1). All but two of the attending participants (*n* = 1155/1157, 99.8%) had been formally discharged from the service at the time of analysis (June 2023).

### Demographics of people with first-time referrals to the ACS (Table 2 and Supplementary Data, Table S5)

**Table 2:** Demographics of first referrals to the Airway Clearance Service (ACS), n (%)

	**All ** ***n*** ** = 1335**	**Attending ** ***n*** ** = 1157**	**Non-attending ** ***n*** ** = 178**
**Gender**			
Male	543 (41)	476 (41)	67 (38)
Female	792 (59)	681 (59)	111 (62)
**Age in years,** mean (SD)	68.98 (13)	69.18 (13)	67.75 (15)
**ARIA classification** ^a^			
Major city	1114 (83)	972 (84)	142 (80)
Inner regional	177 (13)	151 (13)	26 (15)
Outer regional	37 (3)	29 (3)	8 (4)
Remote	7 (1)	5 (0)	2 (1)
Very remote	0	0	0
Multiple classifications	126		
**SES (IRSAD Quintile)**			
0 no SEIFA score^b^	190 (14)	171 (15)	19 (11)
1 most disadvantaged	78 (6)	67 (6)	11 (6)
2	249 (19)	210 (18)	39 (22)
3	252 (19)	217 (19)	35 (20)
4	228 (17)	200 (17)	28 (16)
5 most advantaged	338 (25)	292 (25)	46 (26)
**Referral source**			
SALHN Respiratory Inpatients	69 (5)	48 (4)	21 (12)
SALHN Respiratory Outpatients	576 (43)	497 (43)	79 (44)
CALHN Respiratory Outpatients	4 (1)	3 (0)	1 (0)
Private Respiratory Physician	535 (40)	476 (41)	59 (33)
General Practitioner	58 (4)	54 (5)	4 (2)
Internal Referral^c^	93 (7)	79 (7)	14 (8)
**Condition/reason stated on referral**			
Asthma	247 (19)	213 (18)	34 (19)
Chronic Obstructive Pulmonary Disease	408 (31)	344 (30)	64 (36)
Asthma-COPD Overlap	40 (3)	37 (3)	3 (2)
Bronchiectasis	649 (49)	579 (50)	70 (39)
Interstitial Lung Disease	83 (6)	71 (6)	12 (7)
Tracheobronchomalacia	28 (2)	24 (2)	4 (2)
Chronic cough	52 (4)	44 (4)	8 (4)
Lung cancer	20 (1)	17 (1)	3 (2)
Pneumonia	10 (1)	7 (1)	3 (2)
Other^d^	104 (8)	90 (8)	14 (8)
**Number of conditions/reasons for referral**			
Single	1087 (81)	941 (81)	146 (82)
Multiple	248 (19)	216 (19)	32 (18)

*ARIA* Accessibility/Remoteness Index of Australia, *SES* Socioeconomic Status, *IRSAD* Index of Relative Socio-Economic Advantage and Disadvantage, *SEIFA* Socio-Economic Indexes for Areas, *SALHN* Southern Adelaide Local Health Network, *CALHN* Central Adelaide Local Health Network, *COPD* Chronic Obstructive Pulmonary Disease

^a^First ARIA classification only

^b^Postcode not received a SEIFA score due to low populations or low response rates for certain variables

^c^Referral from within the suite of out of hospital services e.g. Pulmonary Rehabilitation

^d^Aspiration, laryngeal cancer, obstructive sleep apnoea, mucus hypersecretion, pulmonary hypertension, dyspnoea, hemi-diaphragm elevation

More women (*n* = 792/1335, 59%) than men (*n* = 543/1335, 41%) were referred to the ACS with the average age of referees being 69 years. Most resided in major cities (*n* = 1114/1335, 83%) and inner regional areas (*n* = 177/1335, 13%) with near equal distribution across the IRSAD quintiles 2 (disadvantaged) to 5 (most advantaged). There were no differences between those referred who did and did not attend the service in age, gender, geographic location, socio-economic disadvantage, or condition/reason for referral. Referral source was different in people who did or did not attend the service (χ2 = 22.563, *p* = 0.0004) with those referred from public SALHN inpatient wards less likely to attend (21 of 69 referrals from this source did not attend, 30%). The three most common ‘reasons’ for referral to the ACS were bronchiectasis (*n* = 649/1335, 49%), COPD (*n* = 408/1335, 31%) and asthma (*n* = 247/1335, 19%).

### Self-reported information (Supplementary Data, Table S2)

Self-reported symptom information obtained from the ACS registration forms (returned by 789/1157, 68%) showed that daily mucus load was common (*n* = 500/789, 63%), and not only associated with infections (*n* = 468/789, 59%). Mucus was described as thick (*n* = 239/789, 30%), sticky (*n* = 226/789, 29%) and difficult to expectorate (*n* = 456/789, 58%). Symptoms of reflux or heartburn (*n* = 407/789, 52%), sinusitis or rhinitis (*n* = 223/789, 28%), leaking urine (*n* = 267/789, 34%) and musculoskeletal pain (*n* = 223/789, 28%) on coughing were commonly reported.

### Service provision and metrics (Table 3)

**Table 3:** Airway Clearance Service provision and metrics, n (%)

	Discharged participants *n* = 1155
**Time to first appointment in calendar days** ^a^ **, mean (SD)**	36 (24.76)
	Min 0 (same day)
	Max 432
**Number of occasions of service received, mean (SD)**	3 (1.73)
	Max 13
	Min 1
**Length of time in service in calendar days, median (IQR 1–3)**	100 (49–187)
**Discharge summary completed by clinician**	
Yes	869 (75)
No	286 (25)
**Satisfaction survey completed by participant**	
Yes	289 (25)
No	866 (75)
**Device prescribed**	
Bubble PEP	263
Pari PEP S	23
Pari-O-PEP	1
Threshold PEP	6
Threshold IMT	0
Powerbreathe	1
Acapella	127
Aerobika	525
Aeroeclipse	50
Saline inhalation therapy via nebuliser	112
Sinus rinse	131
**On-referrals made**	
Musculoskeletal Physiotherapy	2
Women’s Health Physiotherapy	7
Falls Physiotherapy	3
Occupational therapy	2
Dietetics	10
Psychology	9
Respiratory Nurse	5
Speech Pathologist	66
Heart Failure Nurse	1
My Aged Care	2
Social Work	2
Palliative Care	1
Pulmonary Rehabilitation	116

^a^One thousand one hundred fifty seven referrals with first appointment – two participants ongoing in service

A total of 2996 occasions of service (face-to-face clinic *n* = 2108 (70%), phone *n* = 736 (25%), telehealth *n* = 99 (3%), home visit *n* = 53 (2%)) were delivered to the 1157 patients attending the ACS. On average the initial appointment occurred within 36 calendar days of being referred; people attended three times and were discharged within 146 calendar days. A discharge summary to the GP and referrer was completed for most people (*n* = 869/1155, 75%).

The three most common airway clearance devices that were prescribed and provided were oscillating positive expiratory pressure (PEP) devices; the Aerobika (*n* = 525/1157, 45%), bubble PEP (*n* = 263/1157, 23%) and the Acapella (*n* = 127/1157, 11%). The services most on-referred to were PR (*n* = 116), speech pathology (*n* = 66), dietetics (*n* = 10) and psychology (*n* = 9).

### PROMs and satisfaction surveys

Individuals completed both pre and post impact questionnaires around a third of the time (CAT 33%; QOL-B 35%; DASS-21 22%; LCQ 24%) with at least half of responders reporting an improvement in respiratory symptom outcomes during this time that was consistent with the MCID (CAT 53%; DASS 21 27%; LCQ 50%; QOL-B respiratory domain 61%) (Table 4). There were no observable differences (gender, age, referral source and number of appointments with the service) between those completing PROMS and those that did not (Supplementary Data, Table S7). Satisfaction surveys were returned by 289 individual ACS attendees who were formally discharged from the service (289/1155, 25%). Most participants (> 95%) who responded indicated they agreed (agree or strongly agreed) with each statement; less than 2% indicated neutral responses or disagreement (disagree, strongly disagree) (Fig. 2 and Supplementary Data, Table S3).

**Table 4:** Summary of Patient reported outcome measures (PROM) data before and after participation in the airway clearance service (ACS)

		All completed participant questionnaires		Participants who completed both Pre ACS and Post ACS questionnaires	
		Pre ACS	Post ACS	Difference Pre-post	Number meeting or exceeding MCID
Outcome measure	Domain scores	N (%) Mean (SD)	N (%) Mean (SD)	N (%) Mean (SD)	N (% of those who completed pre and post)
**Leicester Cough Questionnaire (*n* = 1155)** *(MCID 1.3 points higher)*	Total score	775 (67) 14.6 (4.1)	280 (24) 16.4 (3.4)	273 (24) 1.7 (3.3)	137 (50)
**COPD Assessment Tool (*n* = 344)** *(MCID 2 points lower)*	Total score	309 (90) 22.8 (7.4)	107 (35) 16.0 (7.8)	101 (33) 2.7 (7.1)	54 (53)
**Depression Anxiety Stress Scale (*n* = 1155)** *(MCID 5 points lower)*	Total score	716 (62) 14.2 (12.0)	260 (23) 13.1 (11.3)	251 (22) 0.8 (9.4)	68 (27)
**Quality of Life – Bronchiectasis (*n* = 579)** *(MCID – see individual domain)*	Physical (≥ 10.0 points)	378 (65) 49.8 (32.0)	161 (43) 55.8 (34.1)	132 (35) -3.7 (18.3)	63 (48)
	Role functioning (≥ 8.0 points)	378 (65) 54.9 (12.8)	161 (43) 57.6 (12.6)	132 (35) -1.6 (14.2)	52 (39)
	Vitality (≥ 10.0 points)	378 (65) 58.7 (13.9)	161 (43) 60.6 (11.6)	132 (35) -0.1 (14.8)	40 (30)
	Emotion (≥ 7.0 points)	378 (65) 79.5 (21.3)	161 (43) 84.3 (18.3)	132 (35) -3.2 (15.5)	51 (39)
	Social (≥ 9.0 points)	378 (65) 57.5 (26.1)	161 (43) 64.1 (23.0	132 (35) -7.3 (22.8)	71 (54)
	Treatment Burden (≥ 9.0 points)	204^a^ (35) 36.3 (14.9)	127^b^ (22) 34.1 (12.8)	77 (13) 1.9 (20.8)	20 (15)
	Health perception (≥ 8.0 points)	378 (65) 49.8 (14.9)	161 (43) 45.5 (15.3)	132 (35) 1.2 (15.2)	53 (40)
	Respiration (≥ 8.0 points)	378 (65) 60.4 (19.2)	161 (43) 67.3 (15.0)	132 (35) -5.0 (15.6)	81 (61)

Leicester Cough Questionnaire and Depression Anxiety Stress Scale provided to all participants; COPD Assessment Tool provided only to those with a referral diagnosis of COPD; Quality of Life – Bronchiectasis provided only to those with a referral diagnosis of bronchiectasis

*MCID*-Minimum clinically important difference

^a^Missing responses for treatment burden domain questions resulted in a reduced number scored (*n* = 204/378 [54%]) complete domain responses

^b^Missing responses for treatment burden domain questions resulted in a reduced number scored (*n* = 127/161, [79%]) complete domain responses

## Discussion

This evaluation has described an operating ACS, which to our knowledge was the sole community-based outreach clinic for adults with chronic lung conditions and mucus hypersecretion in this region [30]. Airway clearance services in Australia have been described as ad hoc, understaffed, and inaccessible due to a lack of dedicated resources and space [18, 30]. In contrast, the recognised needs of this health network were met with a stand-alone ACS that was easily accessed by consumers, many of whom described an improvement in their health-related quality of life because of their participation in the service.

Participation rates for this ACS were high (87% attendance rate) and timely (within 36 calendar days) comparing more favourably to rates reported of those declining to attend PR (50%) [31] or waiting for PR services (58% of people with stable COPD start PR within 90 days) [32]. The occasions of intervention delivered by the ACS to optimise the participant were low (on average 3 sessions), similar to reports from other specialised, symptom-focused self-management programs such as breathlessness intervention services [33]. The high number of referrals received from the private sector was likely due to the existing affiliations with referring respiratory physicians (who worked in both public and private settings) and may also reflect a lack of private respiratory physiotherapy services in the local area.

Bronchiectasis was the condition most frequently referred to the ACS, which aligned with international clinical guidelines available at the time [10]. With publication (subsequent to 2021 [6, 9]) of position statements on bronchiectasis suggesting intensive airway clearance strategies for those failing antibiotic therapy for an acute exacerbation, and recommending ongoing ambulatory airway clearance review biannually, referral numbers from all sources including acute hospitals may increase in the future. While a significant proportion of the ACS participants had COPD, few referrals overall came from the local hospital respiratory inpatient wards. This may reflect specific guideline recommendations for airway clearance in people with COPD at that time (2017–2021) where referral to ACSs were not mentioned, compared with referral to PR which was established as part of a checklist of recommended interventions in the Australian COPD-X guidelines [11]. A proportion of hospital admissions due to exacerbations of COPD may be potentially preventable by optimised ambulatory care (eg 47% in a study of three Australian hospitals [34]). Non- referral to an ACS could reflect a missed opportunity for the subset of people with COPD also experiencing mucus hypersecretion. Emerging evidence of the impact of mucus hypersecretion on mortality (people with COPD and mucus obstruction had 20% higher mortality rate than those who did not [35]) and reduced exacerbation frequency for those with COPD using airway clearance devices (*n* = 4 studies; rate ratio, 0.81; CI, 0.58–1.12; *p* < 0.05) [36] could translate to more ACS referrals for people with COPD in the future.

One of the unanticipated findings of this evaluation was the number of people living with asthma that were referred. At service planning it was anticipated that only low numbers of people living with asthma would be referred hence the absence of an asthma-specific PROM. The prevalence of self-reported symptoms was different across people with the three most commonly referred conditions (bronchiectasis only *n* = 310, COPD only *n* = 133, asthma only *n* = 68). People referred with a diagnosis of asthma were more likely to report experiences of reflux (χ2 = 15.42, *p* = 0.000), sinus problems (χ2 = 14.41, *p* = 0.001), incontinence (χ2 = 24.05, *p* < 0.000) and musculoskeletal pain on coughing (χ2 = 23.51, *p* < 0.000). People referred with a diagnosis of asthma were more likely to report mucus that was thick (χ2 = 8.99, *p* = 0.011), sticky (χ2 = 13.24, *p* = 0.001) and difficult to expectorate (χ2 = 7.92, *p* = 0.019) (Supplementary data, Table S6).

During the audit timeframe, this ACS was impacted by the Covid-19 pandemic. In South Australia, state borders closed (24th March 2020 to 23rd November 2021) with a variety of isolation orders, social restrictions and lockdowns (November 18, 2020 3 days; July 20, 2021 7 days) throughout 2020 and 2021 affecting service delivery [37]. The ACS continued to provide urgent face-to-face care at the discretion of the respiratory physiotherapists in line with local infection control protocols and consumer preferences. The changes in service delivery saw the proportion of face-to-face appointments reduce (88% in 2019 to 53% in 2020), phone consults increase (10% in 2019 to 45% in 2020) and the introduction of a telehealth platform (Supplementary Data, Table S4).

Airway clearance devices were funded by the ACS and prescribed relatively often. This is consistent with reports from other metropolitan services in Australia [18]. Providing devices at no cost to the client may have contributed to the uptake of the intervention by creating a supportive environment for practicing self-management airway clearance behaviours at home [38]. Actual devices most frequently prescribed (i.e., both commercial and low-cost PEP devices) were the same as those reported in a national survey [18].

This service model offered the voluntary completion of PROMs to all participants in line with the COPDX guidelines (CAT) [11] and recommendations on the Bronchiectasis Toolbox resource webpage (QOL-B, LCQ) [39] respectively. Voluntary completion rates were modest, which limited interpretation of pre-post questionnaire changes as an indicator of clinical improvement. In the context of a clinical service, participants and clinicians may use pre and post PROM data to monitor severity of disease related impairment and the impact of any clinical interventions. Outcome scores could be analysed by clinicians and shared with participants to ensure the information is clinically meaningful, informing practice and contributing to positive behaviour change in the adoption of self-management strategies [38]. Ways to improve the accessibility and completion rates of these questionnaires, such as the use of digital platforms are currently being considered.

On-referral numbers to a respiratory-specialised speech pathologist from the ACS were notably high. The co-location of allied health services at the health site facilitated dual assessment and intervention where possible by physiotherapy and speech pathology for people with chronic refractory cough and inducible laryngeal obstruction type presentations [40]. Structuring the environment in this way improved the accessibility of services and is likely to have supported implementation of a multidisciplinary approach to care.

Throughout the evaluation period, the planned 1.0 FTE of physiotherapy and single site clinics were not sufficient to meet the scope of referrals received. Additional physiotherapy resource and outreach clinic locations were sought and gained in 2019 to ensure the service was able to maintain its level of timely access and frequency of clinical intervention. Since this time (beyond 2021), local community-based health service infrastructure and physiotherapy resources have not expanded to meet the exponential growth (volume and complexity of referrals) seen in the ACS resulting in increased wait times for this highly sought-after service.

### Study limitations

Data collection reflected the practices of the service, not a clinical trial, and assumed that planned administrative processes were adhered to. Planned data analysis for PROMs focussed upon the proportion of participants completing both pre and post outcomes assessments, and meeting or exceeding established MCIDs for specific instruments. Testing for statistical differences pre to post service usage was not planned as completion rates of PROMs was voluntary, completion rates were likely to be less than 80% of possible participants, and a high potential for missing data and/or PROMs. The observed response rate for people submitting PROMS both pre and post ACS (paired data) as a percentage of all pre-ACS PROMS was less than 40% across specific PROMS. As the service plan for data collection and establishment of a database were for the purposes of recording and tracking service provision (e.g., referral source, diagnosis, dates of appointments, budget outlay on devices), not all aspects of clinical intervention delivery or outcomes were recorded. For example, data on the frequency of other common airway clearance interventions such as general education, the Active Cycle of Breathing Technique [41] and forced expiratory techniques were not collected by the ACS, nor reported on in this service evaluation. Similarly, first line education about management of the clients’ self-reported concurrent symptoms (e.g., reflux, heartburn, sinusitis, pain or urine leakage) was not captured in the audit data. It is not possible to interpret service efficacy from pre-service symptom questionnaires, pre-post service PROMs, and post-service satisfaction, as completion was voluntary and there may be unknown differences between those who participated or did not. Diagnosis and referral reason were recorded from the referral and were not confirmed which may not reflect all known respiratory co-morbidities in the cohort. Consumers were not consulted in the early-stage planning phases of this ACS. An analysis of the impact of this service on consumer healthcare usage would provide an important economic viewpoint, that coupled with consumer and healthcare professional insights into the ideal model of service delivery may see this service evolve in the future.

## Conclusion

This report provides a real-life example of an ACS evaluation with potential applications to clinical service planning, implementation, quality improvement and development of quality standards. To align with current recommendations, the provision of physiotherapy-led ACSs alongside multidisciplinary teams caring for people with chronic lung conditions should be explored and prioritised by health services world-wide.

### Supplementary Information

**Additional file 1. Supplementary Data, S1.** Patient Satisfaction Survey.

**Additional file 2: Supplementary Data, Table S2.** Airway Clearance Service Registration forms - self-reported information, n (%).

**Additional file 3: Supplementary Data, Table S3. **Patient Satisfaction Survey Results, *n* = 289 (%).

**Additional file 4: Supplementary Data, Table S4. **Supplementary Data, Table S4: Mode of service delivery data during Covid-19.

**Additional file 5: Supplementary Data, Table S5. **Demographics of first referrals to the Airway Clearance Service (ACS), n (%).

**Additional file 6: Supplementary Data, Table S6. **Self-reported symptoms by single diagnosis.

**Additional file 7: Supplementary Data, Table S7.** Characteristics of participants completing any PROMs, n (%).

## Data Availability

The de-identified dataset analysed during the current study is available for reproducibility reasons from the corresponding author on reasonable request, subject to and with approval of the overseeing Human Research Ethics Committee.

## Abbreviations

COPD: Chronic obstructive pulmonary disease
PR: Pulmonary Rehabilitation
ACS: Airway clearance service
CF: Cystic Fibrosis
SALHN: Southern Adelaide Local Health Network
HREC: Human Research Ethics Committee
FTE: Full-time equivalent
PROMs: Patient-reported outcome measures
LCQ: Leicester Cough Questionnaire
DASS21: Depression Anxiety and Stress Scale
CAT: COPD Assessment Tool
QOL-B: Quality of Life-Bronchiectasis
IRSAD: Index of Relative Socio-Economic Advantage and Disadvantage
ARIA: Accessibility/Remoteness Index of Australia
MCID: Minimal clinically important difference
PEP: Positive expiratory pressure
