# Supplementary material for: Physiotherapy-led, community-based airway clearance services for people with chronic lung conditions: a retrospective descriptive evaluation of an existing model of care
Source: BMC Health Serv Res. 2024 Jan 18;24:98. doi: 10.1186/s12913-024-10550-x (PMC10795339; doi:10.1186/s12913-024-10550-x)
Supplement: Supplementary file 1 — Additional file 1. Supplementary Data, S1. Patient Satisfaction Survey. [file 12913_2024_10550_MOESM1_ESM.docx]

| GP Plus Respiratory Out of Hospital Services |
| --- |
| Consumer Experience Questionnaire – Airway Clearance Program |

Dear

Recently you have been assessed and treated by our Respiratory physiotherapist…………………………………..as part of the Airway Clearance Program.

We value your opinion and insight into helping us to provide a quality service. This questionnaire should take approximately 5 minutes to complete and all responses are strictly confidential. We would appreciate your feedback to enable us to evaluate the program so we can continue to offer the best care possible.

Please tick how strongly you agree or disagree with the following statements

| **Questions** | **Strongly Disagree** | **Disagree** | **Neutral** | **Agree** | **Strongly Agree** |
| --- | --- | --- | --- | --- | --- |
| 1. I have a better understanding of my lung condition |  |  |  |  |  |
| 1. I have a better understanding of ways to clear my secretions |  |  |  |  |  |
| 1. I feel more confident at managing my secretions |  |  |  |  |  |
| 1. I am confident in performing the exercises prescribed to me |  |  |  |  |  |
| 1. I am confident in using the devices prescribed to me |  |  |  |  |  |
| 1. I prefer seeing my Respiratory Physiotherapist in a community setting such as a GP+ Centre rather than in a Hospital setting |  |  |  |  |  |

Is there any way the service can be improved?

## _______________________________________________________________________ ______________________________________________________________________________________________________________________________________________

## Any other comments? _____________________________________________________________________________________________________________________________________________________________________________________________________________________

Thankyou
