# Supplementary material for: Physiotherapy-led, community-based airway clearance services for people with chronic lung conditions: a retrospective descriptive evaluation of an existing model of care
Source: BMC Health Serv Res. 2024 Jan 18;24:98. doi: 10.1186/s12913-024-10550-x (PMC10795339; doi:10.1186/s12913-024-10550-x)
Supplement: Supplementary file 2 — Additional file 2: Supplementary Data, Table S2. Airway Clearance Service Registration forms - self-reported information, n (%). [file 12913_2024_10550_MOESM2_ESM.docx]

| Supplementary Data, Table S2: Airway Clearance Service Registration forms - self-reported information, n (%) | |
| --- | --- |
|  | First referrals attending  n=789 |
| **Ever smoker** |  |
| Yes | 399 (51) |
| No | 388 (49) |
| Not answered | 2 (0) |
| **Current** **smoker** |  |
| Yes | 62 (8) |
| No | 725 (92) |
| Not answered | 2 (0) |
| **Daily sputum load** |  |
| Yes | 500 (63) |
| No | 259 (33) |
| Not answered | 30 (4) |
| **Sputum load with infections only** |  |
| Yes | 191 (24) |
| No | 468 (59) |
| Not answered | 130 (17) |
| **Sputum colour when well** |  |
| Clear | 263 (33) |
| White | 264 (33) |
| Pale yellow | 243 (31) |
| Dark yellow | 42 (5) |
| Green | 59 (7) |
| Brown | 31 (4) |
| Other (pink, grey) | 5 (1) |
| **Sputum colour when unwell** |  |
| Clear | 48 (6) |
| White | 73 (9) |
| Pale yellow | 223 (28) |
| Dark yellow | 202 (26) |
| Green | 227 (29) |
| Brown | 102 (13) |
| Other^1^ | 14 (2) |
| **Amount of sputum per day over past 2 weeks** |  |
| None | 74 (9) |
| ½ tsp | 116 (15) |
| 1 tsp | 111 (14) |
| 1Tbsp | 159 (20) |
| ¼ cup | 89 (11) |
| ½ cup | 47 (6) |
| Other^2^ | 16 (2) |
| Not answered | 177 (22) |
| **Sputum consistency** |  |
| Watery | 52 (7) |
| Loose | 93 (12) |
| Creamy | 133 (17) |
| Thick | 239 (30) |
| Sticky | 226 (29) |
| Stringy | 77 (10) |
| Frothy | 72 (9) |
| **Sputum difficult to expectorate** |  |
| Yes | 456 (58) |
| No | 226 (29) |
| Not answered | 107 (13) |
| **Haemoptysis in the last 6 months** |  |
| Yes | 75 (10) |
| No | 678 (86) |
| Not answered | 36 (4) |
| **Courses of antibiotics taken in previous 12 months** |  |
| None | 149 (19) |
| One | 140 (18) |
| Two | 137 (17) |
| Three | 98 (12) |
| Four | 71 (9) |
| Five or more | 112 (14) |
| Low dose | 13 (2) |
| Not answered | 69 (9) |
| **Experience symptoms of reflux or heartburn** |  |
| Yes | 407 (52) |
| No | 333 (42) |
| Not answered | 49 (6) |
| **Experience symptoms of sinusitis or rhinitis** |  |
| Yes | 223 (28) |
| No | 463 (59) |
| Not answered | 103 (13) |
| **Leak urine when cough or exercise** |  |
| Yes | 267 (34) |
| No | 482 (61) |
| Not answered | 40 (5) |
| **Previously seen a respiratory physiotherapist** |  |
| Yes | 152 (19) |
| No | 576 (73) |
| Not answered | 61 (8) |
| **Joint or muscle pain when coughing** |  |
| Yes | 223 (28) |
| No | 503 (64) |
| Not answered | 63 (8) |
| **Location of pain when coughing** |  |
| Neck | 11 |
| Shoulders | 18 |
| Chest | 129 |
| Abdomen | 29 |
| Back | 61 |
| Other^3^ | 34 |
| Not answered | 19 |
| **Exercise limitations** |  |
| Shortness of breath | 474 |
| Pain | 190 |
| Tiredness | 263 |
| No interest | 36 |
| Other^4^ | 31 |
| Not answered | 114 |
| *^1^ pink, red, grey*  *^2^ 1 cup or more*  *^3^jaw, throat, arms, hands, knees, legs, hips, groin, elbows*  *^4^weather, time, quadriplegia, balance, cough, heart failure, age, hernia, bone density, oxygen saturation* |  |
