# Supplementary material for: Physiotherapy-led, community-based airway clearance services for people with chronic lung conditions: a retrospective descriptive evaluation of an existing model of care
Source: BMC Health Serv Res. 2024 Jan 18;24:98. doi: 10.1186/s12913-024-10550-x (PMC10795339; doi:10.1186/s12913-024-10550-x)
Supplement: Supplementary file 3 — Additional file 3: Supplementary Data, Table S3. Patient Satisfaction Survey Results, n = 289 (%). [file 12913_2024_10550_MOESM3_ESM.docx]

| Supplementary Data, Table S3: Patient Satisfaction Survey Results, n=289 (%) | | | | | | |
| --- | --- | --- | --- | --- | --- | --- |
| **Questions** | **Strongly Disagree** | **Disagree** | **Neutral** | **Agree** | **Strongly Agree** | **Not answered** |
| I have a better understanding of my lung condition | 0  (0%) | 3  (1%) | 8  (3%) | 137  (47%) | 141  (49%) | 0 |
| I have a better understanding of ways to clear my secretions | 0  (0%) | 1  (0%) | 5  (2%) | 117  (40%) | 166  (57%) | 0 |
| I feel more confident at managing my secretions | 1  (0%) | 2  (1%) | 7  (2%) | 138  (48%) | 139  (48%) | 2  (1%) |
| I am confident in performing the exercises prescribed to me | 1  (0%) | 1  (0%) | 8  (3%) | 130  (45%) | 148  (51%) | 1  (1%) |
| I am confident in using the devices prescribed to me | 2  (1%) | 2  (1%) | 7  (2%) | 127  (44%) | 147  (51%) | 4  (2%) |
| I prefer seeing my Respiratory Physiotherapist in a community setting such as a GP+ Centre rather than in a hospital setting (n=244) | 1  (0%) | 3  (1%) | 19  (8%) | 78  (32%) | 143  (59%) | 0  (0%) |
