# Supplementary material for: Physiotherapy-led, community-based airway clearance services for people with chronic lung conditions: a retrospective descriptive evaluation of an existing model of care
Source: BMC Health Serv Res. 2024 Jan 18;24:98. doi: 10.1186/s12913-024-10550-x (PMC10795339; doi:10.1186/s12913-024-10550-x)
Supplement: Supplementary file 4 — Additional file 4: Supplementary Data, Table S4. Supplementary Data, Table S4: Mode of service delivery data during Covid-19. [file 12913_2024_10550_MOESM4_ESM.docx]

Supplementary Data, Table S4: Mode of service delivery data during Covid-19

| **All attended appointments** | **2017** | **2018** | **2019** | **2020** | **2021** |
| --- | --- | --- | --- | --- | --- |
| **Face to face** | 189 (86) | 344 (85%) | 491 (88%) | 448 (53%) | 600 (79%) |
| **Phone** | 11 (5%) | 49 (12%) | 57 (10%) | 377 (45%) | 141 (19%) |
| **Telehealth** | 0 (0%) | 0 (0%) | 0 (0%) | 12 (1%) | 10 (1%) |
| **Home Visits** | 20 (9%) | 12 (3%) | 8 (2%) | 6 (1%) | 6 (1%) |
| **Total** | 220 | 405 | 556 | 843 | 757 |
