# Supplementary material for: Physiotherapy-led, community-based airway clearance services for people with chronic lung conditions: a retrospective descriptive evaluation of an existing model of care
Source: BMC Health Serv Res. 2024 Jan 18;24:98. doi: 10.1186/s12913-024-10550-x (PMC10795339; doi:10.1186/s12913-024-10550-x)
Supplement: Supplementary file 5 — Additional file 5: Supplementary Data, Table S5. Demographics of first referrals to the Airway Clearance Service (ACS), n (%). [file 12913_2024_10550_MOESM5_ESM.docx]

| Supplementary Data, Table S5: Demographics of first referrals to the Airway Clearance Service (ACS), n (%) | | | | |  |
| --- | --- | --- | --- | --- | --- |
|  | **All**  **n=1335** | **Attending**  **n=1157** | | **Non-attending**  **n=178** | **p value** |
| **Gender** |  |  |  | | 0.3761 |
| Male | 543 (41) | 476 (41) | | 67 (38) |  |
| Female | 792 (59) | 681 (59) | | 111 (62) |  |
| **Age in years,** mean (SD) | 68.98 (13) | 69.18 (13) | | 67.75 (15) |  |
| **ARIA classification^1^** |  |  | |  | 0.2387 |
| Major city | 1114 (83) | 972 (84) | | 142 (80) |  |
| Inner regional | 177 (13) | 151 (13) | | 26 (15) |  |
| Outer regional | 37 (3) | 29 (3) | | 8 (4) |  |
| Remote | 7 (1) | 5 (0) | | 2 (1) |  |
| Very remote | 0 | 0 | | 0 |  |
| Multiple classifications | 126 |  | |  |  |
| **SES (IRSAD Quintile)** |  |  | |  | 0.8824 |
| 0 no SEIFA score^2^ | 190 (14) | 171 (15) | | 19 (11) |  |
| 1 most disadvantaged | 78 (6) | 67 (6) | | 11 (6) |  |
| 2 | 249 (19) | 210 (18) | | 39 (22) |  |
| 3 | 252 (19) | 217 (19) | | 35 (20) |  |
| 4 | 228 (17) | 200 (17) | | 28 (16) |  |
| 5 most advantaged | 338 (25) | 292 (25) | | 46 (26) |  |
| **Referral source** |  |  | |  | **0.0004** |
| SALHN Respiratory Inpatients | 69 (5) | 48 (4) | | 21 (12) |  |
| SALHN Respiratory Outpatients | 576 (43) | 497 (43) | | 79 (44) |  |
| CALHN Respiratory Outpatients | 4 (1) | 3 (0) | | 1 (0) |  |
| Private Respiratory Physician | 535 (40) | 476 (41) | | 59 (33) |  |
| General Practitioner | 58 (4) | 54 (5) | | 4 (2) |  |
| Internal Referral^3^ | 93 (7) | 79 (7) | | 14 (8) |  |
| **Condition/reason stated on referral** |  |  | |  | 0.3804 |
| Asthma | 247 (19) | 213 (18) | | 34 (19) |  |
| Chronic Obstructive Pulmonary Disease | 408 (31) | 344 (30) | | 64 (36) |  |
| Asthma-COPD Overlap | 40 (3) | 37 (3) | | 3 (2) |  |
| Bronchiectasis | 649 (49) | 579 (50) | | 70 (39) |  |
| Interstitial Lung Disease | 83 (6) | 71 (6) | | 12 (7) |  |
| Tracheobronchomalacia | 28 (2) | 24 (2) | | 4 (2) |  |
| Chronic cough | 52 (4) | 44 (4) | | 8 (4) |  |
| Lung cancer | 20 (1) | 17 (1) | | 3 (2) |  |
| Pneumonia | 10 (1) | 7 (1) | | 3 (2) |  |
| Other^4^ | 104 (8) | 90 (8) | | 14 (8) |  |
| **Number of conditions/reasons for referral** |  |  | |  | 0.8252 |
| Single | 1087 (81) | 941 (81) | | 146 (82) |  |
| Multiple | 248 (19) | 216 (19) | | 32 (18) |  |
| *ARIA: Accessibility/Remoteness Index of Australia; SES: Socioeconomic Status; SALHN: Southern Adelaide Local Health Network; CALHN: Central Adelaide Local Health Network; COPD: Chronic Obstructive Pulmonary Disease*  *^1^first ARIA classification only*  *^2^postcode not received a SEIFA score due to low populations or low response rates for certain variables*  *^3^referral from within the suite of out of hospital services e.g. Pulmonary Rehabilitation*  *^4^aspiration, laryngeal cancer, obstructive sleep apnoea, mucus hypersecretion, pulmonary hypertension, dyspnoea, hemi-diaphragm elevation* | | | | | |
