# Supplementary material for: Physiotherapy-led, community-based airway clearance services for people with chronic lung conditions: a retrospective descriptive evaluation of an existing model of care
Source: BMC Health Serv Res. 2024 Jan 18;24:98. doi: 10.1186/s12913-024-10550-x (PMC10795339; doi:10.1186/s12913-024-10550-x)
Supplement: Supplementary file 6 — Additional file 6: Supplementary Data, Table S6. Self-reported symptoms by single diagnosis. [file 12913_2024_10550_MOESM6_ESM.docx]

Supplementary Data, Table S6 – Self-reported symptoms by single diagnosis

| **Self-reported symptom** | **Bronchiectasis only**  n=310 (%) | **COPD only**  n=133 (%) | **Asthma only**  n=68 (%) | χ2 | **p** |
| --- | --- | --- | --- | --- | --- |
| Reflux | 158 (51) | 63 (47) | 51 (75) | 15.4177 | 0.000449 |
| Sinus | 85 (27) | 32 (24) | 33 (49) | 14.4139 | 0.000741 |
| Incontinence | 88 (28) | 40 (30) | 40 (59) | 24.0496 | <0.00001 |
| MSK | 73 (24) | 38 (29) | 36 (53) | 23.515 | <0.00001 |
| Thick sputum | 84 (27) | 41 (31) | 31 (46) | 8.9986 | 0.011117 |
| Sticky sputum | 69 (22) | 42 (32) | 29 (43) | 13.2356 | 0.001336 |
| Difficult to expectorate | 165 (53) | 86 (65) | 46 (67) | 7.9247 | 0.019019 |
