# Supplementary material for: Physiotherapy-led, community-based airway clearance services for people with chronic lung conditions: a retrospective descriptive evaluation of an existing model of care
Source: BMC Health Serv Res. 2024 Jan 18;24:98. doi: 10.1186/s12913-024-10550-x (PMC10795339; doi:10.1186/s12913-024-10550-x)
Supplement: Supplementary file 7 — Additional file 7: Supplementary Data, Table S7. Characteristics of participants completing any PROMs, n (%). [file 12913_2024_10550_MOESM7_ESM.docx]

| **Supplementary Data, Table S7**: Characteristics of participants completing any PROMs, n (%) | | | |  |
| --- | --- | --- | --- | --- |
|  | **All**  **n=1335** | **Attending**  **n=1157** | **Any PROM completed^1^**  **n=828** | **No PROM completed**  **n=329** |
| **Gender** |  |  |  |  |
| Male | 543 (41) | 476 (41) | 357 (43) | 119 (36) |
| Female | 792 (59) | 681 (59) | 471 (57) | 210 (64) |
| **Age in years,** mean (SD) | 68.98 (13) | 69.18 (13) | 70.0 (11) | 67.0 (15.2) |
| **Referral source** |  |  |  |  |
| SALHN Respiratory Inpatients | 69 (5) | 48 (4) | 24 (3) | 24 (7) |
| SALHN Respiratory Outpatients | 576 (43) | 497 (43) | 346 (42) | 151 (46) |
| CALHN Respiratory Outpatients | 4 (1) | 3 (0) | 3 (0) | 0 (0) |
| Private Respiratory Physician | 535 (40) | 476 (41) | 371 (45) | 105 (32) |
| General Practitioner | 58 (4) | 54 (5) | 40 (5) | 14 (4) |
| Internal Referral^1^ | 93 (7) | 79 (7) | 44 (5) | 35 (11) |
| **Condition/reason stated on referral** |  |  |  |  |
| Asthma | 247 (19) | 213 (18) | 156 (19) | 57 (17) |
| Chronic Obstructive Pulmonary Disease | 408 (31) | 344 (30) | 240 (29) | 104 (32) |
| Asthma-COPD Overlap | 40 (3) | 37 (3) | 27 (3) | 10 (3) |
| Bronchiectasis | 649 (49) | 579 (50) | 432 (52) | 147 (45) |
| Interstitial Lung Disease | 83 (6) | 71 (6) | 55 (7) | 16 (5) |
| Tracheobronchomalacia | 28 (2) | 24 (2) | 20 (2) | 4 (1) |
| Chronic cough | 52 (4) | 44 (4) | 33 (4) | 11 (3) |
| Lung cancer | 20 (1) | 17 (1) | 11 (1) | 6 (2) |
| Pneumonia | 10 (1) | 7 (1) | 4 (0) | 3 (1) |
| Other^2^ | 104 (8) | 90 (8) | 53 (6) | 37 (11) |
| **Number of appointments,** mean (SD) |  |  |  |  |
|  | - | 2.8 (1.8) | 2.9 (2.0) | 2.8 (1.8) |
| **Device prescription** |  |  |  |  |
|  | - | 1239^3^ | 912 (74) | 327 (26) |
| *PROM: Patient reported outcome measure; SALHN: Southern Adelaide Local Health Network; CALHN: Central Adelaide Local Health Network; COPD: Chronic Obstructive Pulmonary Disease*  *^1^participant has completed at least one of the available four PROMs (pre or post)*  *^1^referral from within the suite of out of hospital services e.g. Pulmonary Rehabilitation*  *^2^aspiration, laryngeal cancer, obstructive sleep apnoea, mucus hypersecretion, pulmonary hypertension, dyspnoea, hemi-diaphragm elevation*  *^3^All devices (Bubble PEP, Pari PEP S, Pari-O-PEP, Threshold PEP, Threshold IMT, Powerbreathe, Acapella, Aerobika, Aeroeclipse, Saline inhalation therapy via nebuliser, Sinus rinse)* | | | |  |
